# Intraoperative indocyanine green fluorescence imaging and multimodal deep learning for predicting non-salvage outcomes in pediatric testicular torsion

**DOI:** 10.3389/fped.2026.1936322

**Published:** 2026-10-28

**Authors:** Zheng Fang, Zhongxu Wang, Xingquan Tu, Yun Zhou, Ting Zhang, Mingcui Fu, Mingliu Huang, Lei Zhang, Xu Cao, Xiangming Yan, Shu Dai

**Affiliations:** Department of Urology, Children’s Hospital of Soochow University, Suzhou, Jiangsu, China

**Keywords:** deep learning, indocyanine green, near-infrared fluorescence imaging, pediatric testicular torsion, pediatric urology, prediction model, testicular salvage

## Abstract

**Introduction:**

Intraoperative assessment of testicular viability in pediatric torsion remains subjective. We evaluated a multimodal indocyanine green near-infrared fluorescence (ICG-NIRF) deep learning model for predicting a composite non-salvage outcome.

**Methods:**

This single-center retrospective analysis used prospectively collected images from consecutive boys with surgically confirmed torsion who underwent emergency exploration and ICG-NIRF imaging. Image collection began in March 2022, and model development was undertaken in January 2025. The endpoint was orchiectomy or ultrasound-confirmed atrophy after orchiopexy, defined as an affected-to-contralateral testicular volume ratio <50% at 6 months. Patients were divided into development (*n* = 107) and internal evaluation (*n* = 46) cohorts. A traditional logistic model was compared descriptively with a two-stream model using operative photographs and standardized 30-s fluorescence images.

**Results:**

Among 153 patients, 52 (34.0%) reached the endpoint. In internal evaluation, the traditional model achieved an area under the receiver operating characteristic curve of 0.889 and a Brier score of 0.182. The multimodal model achieved an area under the curve of 0.946 and a Brier score of 0.128.

**Conclusion:**

Multimodal ICG-NIRF deep learning may provide an objective adjunct for intraoperative decision-making in pediatric testicular torsion. External validation is required.

## Introduction

Acute testicular torsion is a time-critical condition in which the operative decision has implications for pediatric emergency surgery (1–3). After detorsion, surgeons must decide whether the testis is viable enough to preserve, but gross color recovery and bleeding are imperfect surrogates for microcirculatory reperfusion (2, 3).

Indocyanine green near-infrared fluorescence (ICG-NIRF) imaging can visualize perfusion during surgery and may add functional information to conventional inspection (4–6). Existing pediatric torsion reports suggest feasibility, but fluorescence patterns remain semiquantitative and difficult to integrate with the anatomical appearance of the testis during emergency surgery (5–7).

Clinical prediction models and machine learning methods have been explored for testicular salvage, but most available models rely on clinical or preoperative variables rather than paired intraoperative anatomical and fluorescence images (8–13). Because surgical decision-support tools should be interpretable to clinicians (14), we developed a multimodal model that links gross anatomical appearance with ICG-NIRF microcirculation and generates an individualized salvage prediction score intended to support, rather than replace, surgical judgment.

## Materials and methods

Study design and patients. This single-center study retrospectively analyzed prospectively collected intraoperative images from consecutive children who underwent emergency scrotal exploration for suspected acute testicular torsion at a tertiary pediatric urology center in China. Standardized image collection began in March 2022, and retrospective analysis and prediction-model development were undertaken in January 2025. ICG-NIRF imaging was part of routine clinical care. The research protocol was approved before the study analysis by the institutional ethics committee (approval No. 2025CS006), and written informed consent was obtained from the legal guardians. Reporting followed STROBE principles and was informed by reporting recommendations for machine-learning applications in urology (15).

Eligible patients were aged 28 days to 18 years, had clinical and Doppler ultrasonographic suspicion of torsion, underwent emergency exploration with surgically confirmed torsion, received intraoperative ICG-NIRF assessment, and had at least 6 months of follow-up when the testis was preserved. Prespecified exclusions were neonatal torsion, bilateral torsion, torsion of an undescended testis, congenital absence or dysplasia of the contralateral testis, previous surgery for ipsilateral or contralateral torsion, unknown symptom onset, a history of abnormal hepatic or renal function or development, successful preoperative manual detorsion, incomplete clinical data, or loss to follow-up. All otherwise eligible patients underwent ICG-NIRF imaging.

Surgical and fluorescence assessment. After induction of general anesthesia, the affected testis was exposed through an inguinal or transverse scrotal incision. The torsion angle was recorded before immediate detorsion. The testis was wrapped in warm saline gauze for 10 min, and gross color recovery was assessed. ICG was then injected intravenously at 0.2 mg/kg, and near-infrared images were recorded using a 4KIR08 system (Scivita Medical Technology Co., Ltd., Suzhou, China). The model used a static image obtained 30 s after injection. Gross color was graded as 1, pink; 2, mottled pink; 3, light blue; 4, dark blue; 5, black; and 6, hemorrhagic/necrotic. Fluorescence class A indicated both tunical vascular and parenchymal fluorescence, class B tunical vascular fluorescence without parenchymal fluorescence, and class C neither before incision. After incision, C1 indicated bleeding with ICG extravasation, C2 bleeding without extravasation, and C3 neither bleeding nor extravasation. Operative decisions integrated gross appearance, bleeding, fluorescence findings, and surgeon judgment. The operative workflow and visual grading scheme are summarized in Figure 1.

**Figure 1:**
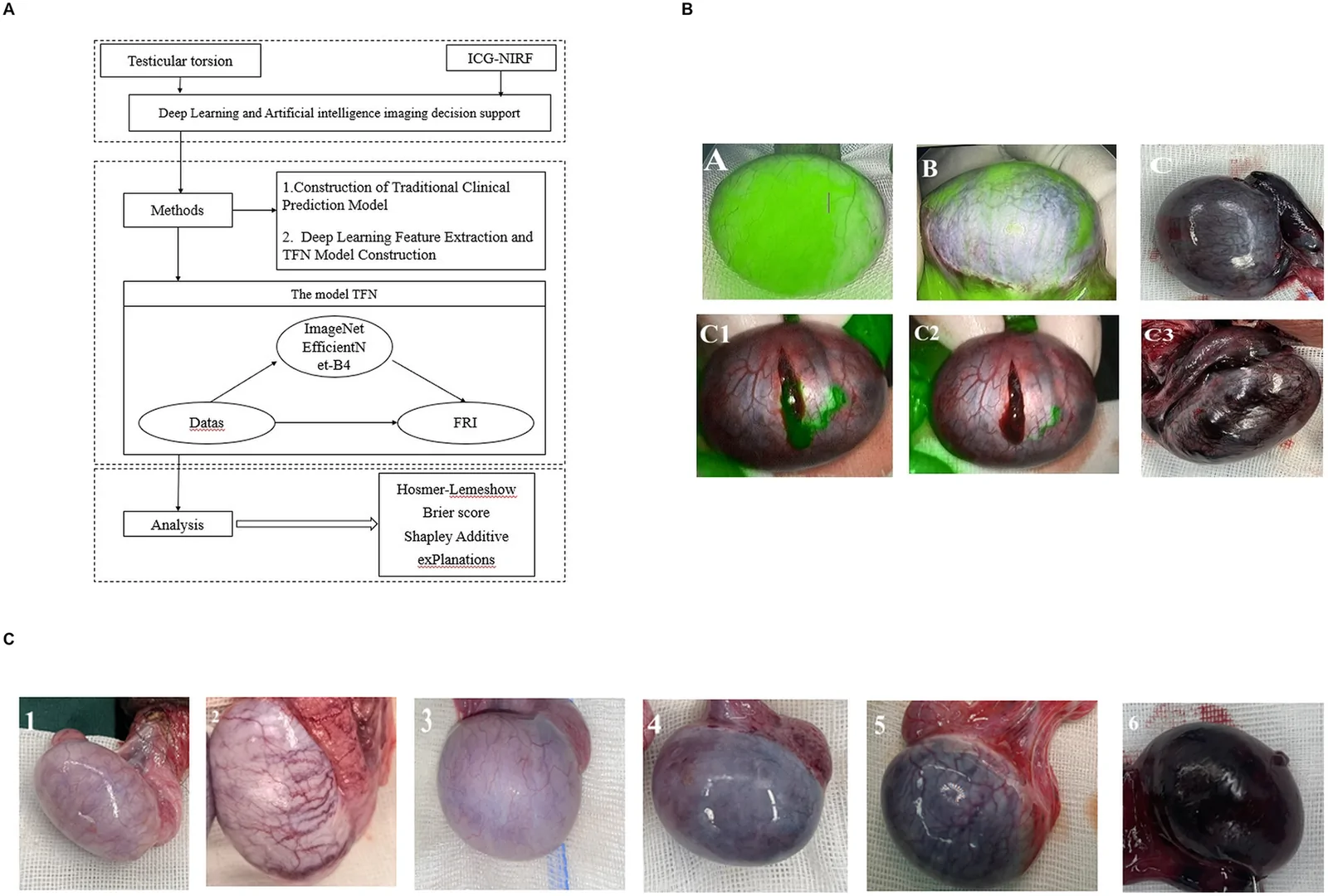
Intraoperative assessment workflow and representative clinical image-labeling framework. **(A)** Testicular torsion and paired ICG-NIRF inputs are prepared as clinical variables and paired operative images; traditional clinical and multimodal fusion models undergo internal evaluation using receiver operating characteristic analysis, Brier score, decision-curve analysis, and Shapley additive explanations. **(B)** Representative ICG-NIRF fluorescence patterns labeled A, B, C, C1, C2, and C3. **(C)** Representative gross testicular color grades 1-6 after detorsion.

Outcome definition. Successful salvage was defined as preservation of the affected testis without atrophy at 6 months. The composite non-salvage endpoint comprised orchiectomy at the index operation or atrophy after orchiopexy. Ultrasound follow-up was performed at 1, 3, and 6 months, with a ± 7-day window at each visit. Bilateral testicular length, width, and height were measured, and volume was calculated using the Lambert formula, V=0.71×length×width×height. Atrophy was defined as an affected-to-contralateral testicular volume ratio <50% at the 6-month assessment. Ultrasound assessors were blinded to group allocation. This composite endpoint reflects both immediate non-preservation and delayed failure after attempted preservation.

Traditional model. Candidate intraoperative variables were screened in the development cohort. Variables with *P* < 0.05 in univariable analysis were entered into forward stepwise multivariable logistic regression. The final traditional model used torsion angle and fluorescence classification, with classes A, B, and C coded as 1, 2, and 3; C1–C3 were combined as class C. This model was retained as a clinically interpretable baseline because both variables are available during emergency exploration.

Image preprocessing and patient-level data partitioning. Each patient contributed one representative intraoperative color photograph and one paired 30-s ICG-NIRF image. All images from the same patient were assigned to the same cohort to avoid data leakage. The cohort was split at the patient level into development (*n* = 107) and internal evaluation (*n* = 46) cohorts at a 7:3 ratio by stratified random sampling according to surgical year, with random seed 202501. Images were de-identified, cropped to the operative field, resized to the required network input size, and normalized with ImageNet mean and standard deviation values.

Deep learning model and training. The anatomical stream used a ResNet-50 backbone pretrained on ImageNet, with a squeeze-and-excitation module and feature pyramid fusion to represent necrotic area, color homogeneity, and the congested-normal tissue boundary. The fluorescence stream used an EfficientNet-B4 backbone to represent vessel fluorescence density, extravasation intensity, and perfusion uniformity. The 512-dimensional anatomical and 256-dimensional fluorescence representations were concatenated and fused through a 768→256→64 fully connected sequence followed by sigmoid output. The model-derived salvage score was termed the Flow Recovery Index (FRI). During training only, augmentation included random rotation within 10 degrees, horizontal flipping, mild brightness and contrast jittering, random cropping, and Gaussian noise. Internal evaluation images were not augmented. Class imbalance was handled using weighted binary cross-entropy loss. The fusion network was trained with the Adam optimizer, an initial learning rate of 1 × 10⁻⁴, mini-batch size 16, early stopping on validation loss, and a maximum of 100 epochs. The same 46-patient cohort was used for early stopping and internal performance evaluation; no separate tuning dataset was reserved. The architecture is shown in Figure 2.

**Figure 2:**
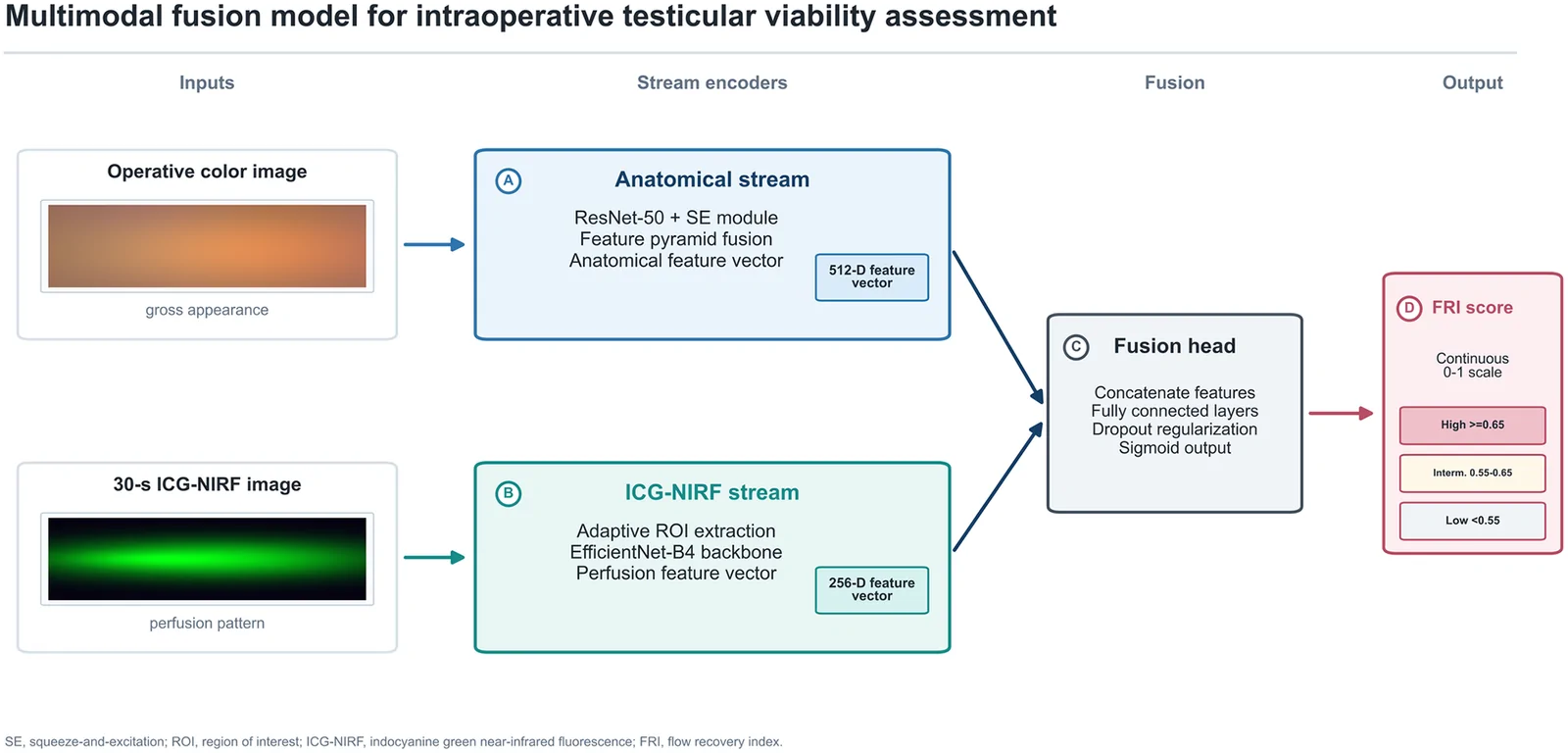
Architecture of the multimodal fusion model. The anatomical stream extracts gross visual features from operative photographs, and the microcirculation stream extracts fluorescence features from paired standardized 30-s ICG-NIRF images. Concatenated features are processed by fully connected layers to generate a continuous salvage prediction score, termed the Flow Recovery Index (FRI). No clinical decision threshold was applied. SE, squeeze-and-excitation; ROI, region of interest.

Statistical analysis. Continuous variables are reported as mean (standard deviation) or median (interquartile range), as appropriate; categorical variables are reported as number and percentage. Between-cohort comparisons used the independent-samples t test or Mann–Whitney U test for continuous variables and the chi-square or Fisher exact test for categorical variables. Patients with incomplete key predictors, outcome data, or follow-up were excluded according to the prespecified eligibility criteria; no imputation was performed. Discrimination was assessed by area under the receiver operating characteristic curve (AUC). Overall predictive accuracy was summarized using the Brier score. Decision curve analysis explored clinical net benefit across threshold probabilities (16). Between-model AUCs were compared descriptively, and no formal statistical superiority claim was made. Two-sided *P* < 0.05 was considered statistically significant for conventional regression analyses.

SHAP analysis. Six image-derived descriptors were examined: necrotic-area proportion, boundary gradient, tissue-color homogeneity, vessel fluorescence density, ICG extravasation intensity, and perfusion uniformity. Anatomical descriptors were calculated as the necrotic-to-total area ratio, gradient at the congested-normal boundary, and local-entropy-based color homogeneity. Adaptive thresholding identified tunical, parenchymal, and peripheral fluorescence regions, from which the fluorescent-pixel proportion, intensity standard deviation, and mean peripheral fluorescence gradient were calculated, respectively. The reference vector comprised the development-set median of each descriptor. Positive and negative SHAP values indicated contributions that increased and decreased the predicted probability of successful salvage, respectively. Global importance was ranked by mean absolute SHAP values.

## Results

Patient cohort. Of 189 children admitted with suspected torsion during the study period, 153 met the eligibility criteria and were included; all had surgically confirmed torsion and underwent ICG-NIRF imaging. The median age was 13.40 years. Orchiectomy was performed in 38 patients (24.8%), and orchiopexy was performed in 115 patients (75.2%). Among the orchiopexy patients, 14 developed testicular atrophy during follow-up. Overall, 52 patients (34.0%) reached the composite non-salvage endpoint and 101 (66.0%) had salvage success. No ICG-related adverse events or imaging failures occurred (0/153 for each). Baseline characteristics are summarized in Table 1, and participant selection in Supplementary Figure S1.

**Table 1:** Baseline characteristics of the development and internal evaluation cohorts.

Characteristic	Overall (*n* = 153)	Development (*n* = 107)	Internal evaluation (*n* = 46)	P value
Age, years	13.40 (12.60–14.10)	13.40 (12.65–14.00)	13.40 (11.77–14.17)	0.900
Weight, kg	44.30 (39.80–49.40)	44.30 (39.95–49.40)	44.80 (39.45–49.20)	0.741
Symptom duration, h	16.00 (9.00–23.00)	16.00 (9.00–23.00)	16.50 (9.50–22.75)	0.731
Torsion angle, degrees	540 (360–720)	540 (360–720)	540 (360–675)	0.674
Fluorescence classification	2 (1–3)	2 (1–3)	2 (1.25–3)	0.373
Non-salvage outcome	52 (34.0%)	34 (31.8%)	18 (39.1%)	0.378

Continuous variables and fluorescence codes are median (interquartile range); outcomes are n (%). Fluorescence classes A, B, and C were coded as 1, 2, and 3. Between-cohort P values were obtained using the Mann–Whitney U test for continuous and ordinal variables and the chi-square test for the outcome.

Traditional intraoperative model. In the development cohort, the non-salvage group had longer symptom duration, greater torsion angle, worse color grade, and worse ICG fluorescence classification. In multivariable analysis, torsion angle (OR 1.01, 95% CI 1.00-1.01; *P* = 0.010) and ICG fluorescence classification (OR 79.03, 95% CI 8.83-707.36; *P* < 0.001) remained independent predictors (Table 2).

**Table 2:** Univariable and multivariable predictors in the development cohort.

Variable	Success (*n* = 73)	Non-salvage (*n* = 34)	Univariable OR (95% CI, P)	Multivariable OR (95% CI, P)
Symptom duration, h	15.9 ± 9.5	21.3 ± 11.6	1.05 (1.01–1.09, *P* = 0.016)	1.06 (0.97–1.17, *P* = 0.212)
Torsion angle, degrees	421.6 ± 180.5	651.2 ± 177.3	1.01 (1.00–1.01, *P* < 0.001)	1.01 (1.00–1.01, *P* = 0.010)
Color grade	3.6 ± 1.0	5.0 ± 0.9	5.69 (2.88–11.28, *P* < 0.001)	1.43 (0.43–4.75, *P* = 0.559)
Fluorescence classification	1.7 ± 0.6	2.9 ± 0.2	100.66 (19.33–524.18, *P* < 0.001)	79.03 (8.83–707.36, *P* < 0.001)

Values in the outcome groups are mean ± standard deviation. Color grades and fluorescence classes were entered as ordinal numerical codes.

OR, odds ratio; CI, confidence interval.

Internal evaluation performance. The traditional model achieved an AUC of 0.913 in the development cohort and 0.889 (95% CI 0.83-0.94) in the internal evaluation cohort. In the internal evaluation cohort, the Brier score was 0.182. The multimodal model achieved an AUC of 0.971 in the development cohort and 0.946 (95% CI 0.91-0.98) in the internal evaluation cohort, with a Brier score of 0.128 (Table 3; Figure 3). These comparisons are descriptive and should not be read as evidence of statistically tested superiority.

**Table 3:** Descriptive performance of the traditional clinical model and multimodal fusion model.

Model	Cohort	AUC (95% CI)	Brier score
Traditional clinical model	Development	0.913	Not reported
Traditional clinical model	Internal evaluation	0.889 (0.83–0.94)	0.182
Multimodal fusion model	Development	0.971	Not reported
Multimodal fusion model	Internal evaluation	0.946 (0.91–0.98)	0.128

AUCs are summarized descriptively; no formal statistical superiority claim is made.

**Figure 3:**
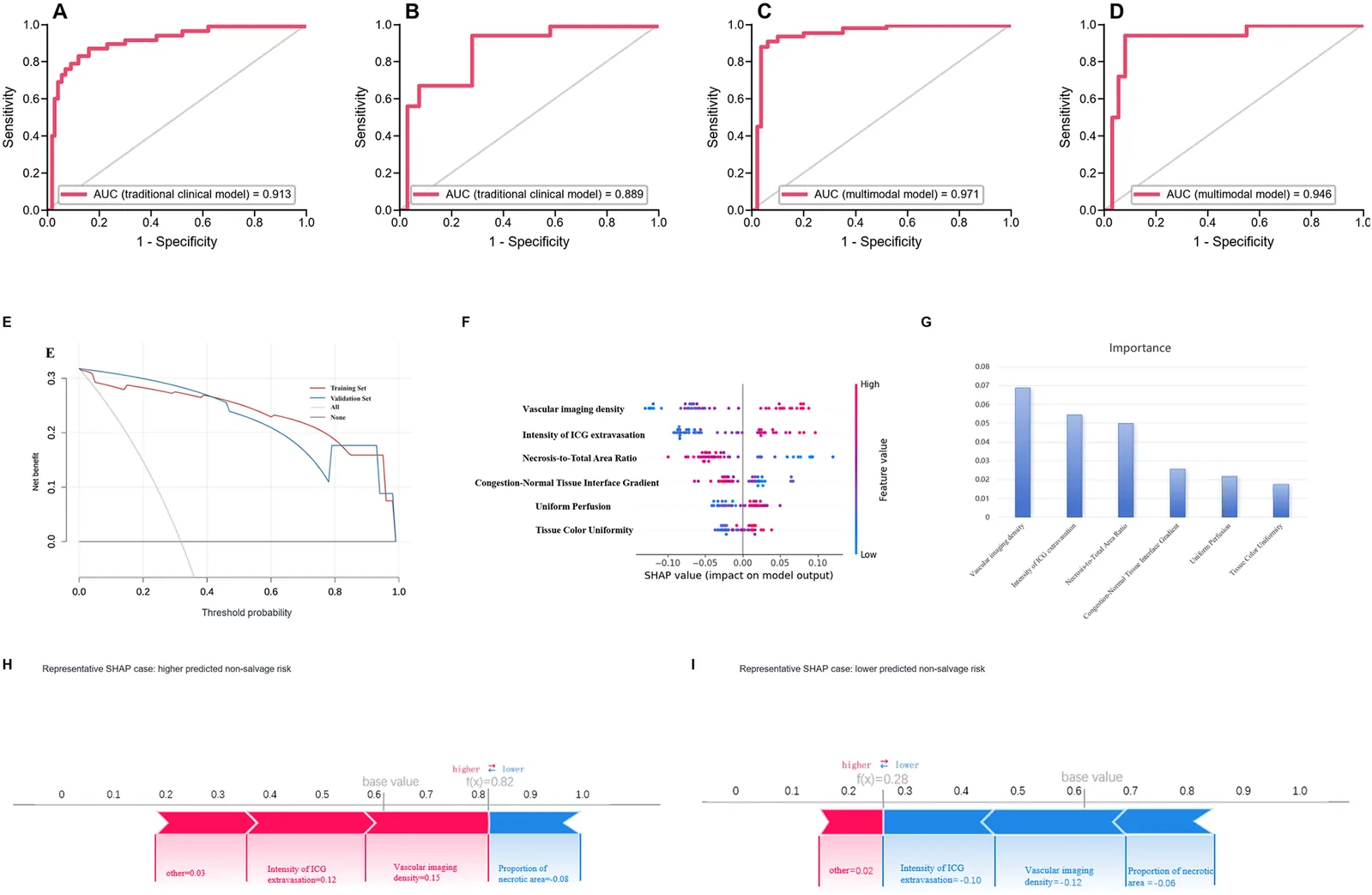
Development, internal evaluation performance, and interpretability. **(A)** Traditional clinical model in the development cohort. **(B)** Traditional clinical model in the internal evaluation cohort. **(C)** Multimodal model in the development cohort. **(D)** Multimodal model in the internal evaluation cohort. **(E)** Decision curve analysis of the multimodal model in the development and internal evaluation cohorts. **(F)** SHAP summary plot for the predicted probability of successful testicular salvage; positive values increase and negative values decrease this probability. **(G)** Mean absolute SHAP feature importance. **(H,I)** Representative SHAP force plots for higher and lower predicted salvage probabilities, respectively. ROC, receiver operating characteristic; AUC, area under the ROC curve; SHAP, Shapley additive explanations.

Interpretability and clinical signal. SHAP analysis identified vessel fluorescence density, ICG extravasation intensity, and necrotic area as the strongest contributors to the predicted probability of successful testicular salvage. These variables are clinically plausible because they reflect microcirculatory recovery, vascular leakage, and gross tissue injury. Representative SHAP cases showed that low fluorescence density and broad necrotic area pushed predictions toward non-salvage, whereas preserved vessel fluorescence and limited necrosis pushed predictions toward salvage (Figure 3).

## Discussion

After detorsion, surgeons must decide whether a compromised testis should be preserved. In this single-center cohort, a multimodal model combining anatomical appearance and ICG-NIRF perfusion showed favorable internal evaluation performance and clinically plausible feature attributions. This finding warrants external evaluation of fluorescence-informed decision support, but does not support standalone clinical use or a claim of model superiority.

The findings are consistent with prior reports suggesting that ICG-NIRF imaging can reveal perfusion differences that are not fully captured by gross inspection (4–7). They also extend earlier prediction-model work in testicular torsion by using paired intraoperative images rather than only clinical variables (11–13). The SHAP contributors are aligned with surgical reasoning: vessel fluorescence density reflects reperfusion, extravasation may reflect vascular injury, and necrotic area reflects gross tissue injury.

Several design choices constrain interpretation. The patient-level split was used to avoid image leakage, augmentation was limited to the development set, and no imputation was performed for missing key data. The internal evaluation cohort also informed early stopping, so the performance estimates may be optimistic. No model-derived clinical decision threshold was used in this study; any future operating threshold would require external validation.

External validation plan. Before clinical deployment, we plan to optimize and test the model in larger prospective multicenter cohorts using prespecified inclusion criteria, standardized ICG dose and imaging timing, centralized image quality control, and site-stratified calibration assessment. Model optimization and final validation will use separate data, with the model locked before final evaluation. The validation study will report discrimination, calibration, decision curves, inference time, failure cases, and subgroup performance by symptom duration, torsion angle, and fluorescence class.

This single-center study used internal evaluation only, so performance may shift across hospitals, imaging systems, surgeons, and patient populations. The number of outcome events was modest relative to the complexity of a high-capacity neural network, and the fluorescence effect estimate was imprecise. Eligibility restrictions and exclusion of patients with incomplete data or follow-up may introduce selection bias. The model used a static 30-s ICG image and did not capture full fluorescence kinetics. Finally, the composite endpoint combines immediate orchiectomy with delayed atrophy after orchiopexy, which is clinically meaningful but biologically heterogeneous. Because operative findings contributed to treatment selection, the model may also reflect surgical decision-making.

In conclusion, multimodal fusion of intraoperative testicular appearance and ICG-NIRF perfusion imaging may support prediction of salvage outcomes and help identify children likely to achieve successful testicular preservation. The model is best viewed as an investigational decision-support adjunct until larger multicenter validation establishes transportability and clinical utility.

## Data Availability

The original contributions presented in the study are included in the article/Supplementary Material, further inquiries can be directed to the corresponding authors.
